# HIV Testing and Counselling in Colombia: Local Experience on Two Different Recruitment Strategies to Better Reach Low Socioeconomic Status Communities

**DOI:** 10.1155/2014/803685

**Published:** 2014-01-30

**Authors:** Jaime Galindo-Quintero, Hector Fabio Mueses-Marin, David Montaño-Agudelo, María Virginia Pinzón-Fernández, Inés Constanza Tello-Bolívar, Beatriz Eugenia Alvarado-Llano, Jorge Luis Martinez-Cajas

**Affiliations:** ^1^Corporación de Lucha Contra el Sida (CLS), Grupo Educación y Salud en VIH/SIDA, Carrera 56 2-120, Cali, Colombia; ^2^The Canada-Colombia Collaboration against HIV/AIDS (CCC-HIV), Carrera 56 2-120, Cali, Colombia; ^3^Faculty of Health Sciences, Universidad del Cauca, Calle 5 4-70, Popayán, Colombia; ^4^Department of Public Health Sciences, Queen's University, Carruthers Hall, Kingston, ON, Canada; ^5^Division of Infectious Diseases, Department of Medicine, Queen's University, Etherington Hall, Kingston, ON, Canada K7L 3N6

## Abstract

HIV testing rates remain very low in Colombia, with only 20% of individuals at risk ever tested. In order to tackle this issue, the Corporacion de Lucha Contra el Sida (CLS) has implemented a multidisciplinary, provider-initiated, population-based HIV testing/counselling strategy named BAFI. In this report, we describe the experience of CLS at reaching populations from low socioeconomic backgrounds in 2008-2009. Two different approaches were used: one led by CLS and local health care providers (BAFI-1) and the other by CLS and community leaders (BAFI-2). Both approaches included the following: consented HIV screening test, a demographic questionnaire, self-reported HIV knowledge and behaviour questionnaires, pre- and posttest counselling, confirmatory HIV tests, clinical follow-up, access to comprehensive care and antiretroviral treatment. A total of 2085 individuals were enrolled in BAFI-1 and 363 in BAFI-2. The effectiveness indicators for BAFI-1 and BAFI-2, respectively, were HIV positive-confirmed prevalence = 0.29% and 3.86%, return rate for confirmatory results = 62.5% and 93.7%, return rate for comprehensive care = 83.3% and 92.8%, and ART initiation rate = 20% and 76.9%. Although more people were reached with BAFI-1, the community-led BAFI-2 was more effective at reaching individuals with a higher prevalence of behavioural risk factors for HIV infection.

## 1. Introduction

According to national statistics there are approximately 150,000 people living with HIV infection in Colombia, an estimated prevalence of 0.57% among those aged 15 to 49 years old [1]. The HIV mortality rate in the year 2010 was estimated to be 5.34/100,000 people, which is similar to the mortality rate in north America at the end of the 90's [2]. In Colombia, HIV testing has not been broadly promoted; individuals traditionally have been expected to voluntarily seek testing, with the exception of prenatal care programs that consistently recommend HIV testing to pregnant women. Provider-initiated strategies for the general population are not the standard of care in Colombia. The current cost of HIV testing in Colombia is high for both HIV screening and confirmatory testing, approximately 10–20 USD and 80–100 USD, respectively. HIV testing can only be performed in accordance with official national regulations, which require informed consent and pre- and posttest counselling that must be delivered by a certified health provider [3]. In addition, conventional HIV testing can only be performed by credentialed clinical microbiologists in private and public laboratories, resulting in limited access to HIV testing in community settings.

Data from the National Health Survey conducted in 2007 showed that the proportion of the general population ever tested for HIV infection remains very low (20%) [4]. Moreover, about half of individuals tested had never received pretest counselling, and more than half had never received post-test counselling [4]. In this regard, the National Health Institute estimates that for every diagnosed HIV positive case there could be eight undiagnosed individuals who are unaware of their status [4]. Some population subgroups appear to be facing more access barriers to HIV-testing. Current HIV counselling and testing practices are not reaching individuals who are less educated, have low-income and no health insurance, and reside in either rural or marginal urban areas and individuals who are elderly [4–6]. In fact, previous studies in Colombia have shown that people from low-income neighborhoods in large urban centers are at higher risk of being infected with HIV (prevalence between 1.97% and 3.86%) [5, 6] than the general population (prevalence rate 0.57%) [1]. Access to HIV testing is hampered by a variety of factors such as stigma, fear of discrimination, low-risk perception of acquiring HIV infection, poor HIV knowledge, and the cost of the test [5, 6]. Therefore, there is a need to increase access to HIV testing in Colombia through either outreach clinics or community strategies with focus on socioeconomically disadvantaged communities.

This need falls under the scope of interest of the *Corporación de Lucha Contra el SIDA* (CLS) which is a nonprofit, multipartner organization that provides comprehensive care to people living with HIV/AIDS and has a mandate to promote HIV-related education and research. Hence, with the purpose of increasing access to HIV counselling and testing (HCT) among low-socioeconomic groups the CLS has developed a multidisciplinary, provider-initiated, HIV testing and counselling strategy named BAFI (from the Spanish *Búsqueda Activa Focal Integral*) that follows the World Health Organization guidelines on HIV testing and counselling [7]. In contrast to what is currently practiced in Colombia for HCT, BAFI uses two strategies that actively seek out target groups for HIV testing. One strategy emphasizes HCT campaigns in health care centers and through health care personnel (BAFI-1). The second strategy was inspired by previous experiences showing that community-based approaches reach people with high-risk behaviors for HIV infection very successfully and can facilitate counselling, improve knowledge, and induce behavioral changes [8]. This strategy emphasizes HCT through the support and invitation of community leaders (BAFI-2).

The main objective of this report is to present the effectiveness of BAFI at reaching population groups of low-socioeconomic status and to compare the performance of these two strategies in providing HCT. The performance of both strategies is assessed using the indicators described on the WHO Guide for Monitoring and Evaluating HIV Testing and Counselling Programmes [9].

## 2. Materials and Methods

### 2.1. Context of BAFI

CLS is a nonprofit organization located in the southwestern Colombian city of Cali that has been committed to providing HIV care since the early years of the epidemic. BAFI was implemented in two cities, Cali and Popayan, with total populations of 2.2 million and 0.26 million, respectively. It is estimated that 48% of the Cali population and 64% of the Popayan population have subsidized health care, indicating that these individuals belong to the lowest socioeconomic strata.

### 2.2. Target Population

BAFI targeted low-income neighbourhoods, defined as communities, where the majority of individuals suffered any or all of the following conditions: (a) houses with inadequate walls, floors or ceilings, (b) lack of at least one public utility, (c) overcrowding (3 or more people per room), (d) high dependency, that is, more than three people depending on a low-educated (two or fewer years of schooling) head of household, and (e) at least one child of school age in the household not attending school.

### 2.3. Recruiting Strategies

BAFI-1 started by establishing contact with local health authorities who manage the public network of health services, ESES (by its acronym in Spanish), which comprises health facilities (ranging from health posts to hospitals) located across Cali and serves the most deprived population groups. The area of influence of each health center varies from one to several neighbourhoods depending on the center's level of complexity. After establishing an agreement with local authorities, representatives including nurses, psychologists, and social workers from 16 health facilities met at CLS for one day to receive training on HIV testing and counselling, as well as research ethics and questionnaire administration. Representatives from all the *comunas* (administrative divisions of cities in Colombia) were present since participating health facilities were distributed across the entire city. Subsequently, each health center autonomously determined how to run the campaign in its neighbourhood. Most health centers chose to place adverts in waiting rooms, corridors, and on bulletin boards, thereby allowing everyone visiting the center access to information about the HCT campaign.

BAFI-2 was established in a different manner. Neighbourhoods in Popayan (*n* = 33) with the lowest socioeconomic status, which housed people displaced by armed conflict and had a high rate of psychoactive drug use and commercial sex, were selected. Approximately 62% of the total population of Popayan live in such neighbourhoods. A peer-to-peer approach was preferred, in which local community leaders, who themselves reside in these low-income neighbourhoods, were contacted and trained in the recruitment methods and sexual health education activities required for HIV counselling. Two males and two females community leaders aged 25–40 were selected based on their respect and trust within, and knowledge of their communities. They were instructed to recruit individuals who, by virtue of their low-socioeconomic status, were considered in need of sexual health services including HIV testing. These potential participants were given information about a local health center where they could receive counselling and testing.

### 2.4. Sample Size

A total of 2458 participants were enrolled through a nonprobabilistic sampling procedure. Potential participants were conveniently recruited either by health centers or by peer facilitators as part of the HIV counselling and testing campaigns in low-income neighbourhoods in both cities. Individuals were recruited until local resources were drained. Hence, the sample size was limited by available funding. Posthoc calculation of this sample renders a precision of 0.3% for the calculation of a prevalence of 0.8% with a confidence interval of 95%.

### 2.5. Description of the Intervention

Both BAFI strategies used the same interventions. First, informed consent was obtained for HIV testing and the administration of a structured questionnaire (SQ). The SQ assessed demographics including age, sex, and educational level, as well as the following HIV risk behaviours: frequency and quantity of alcohol consumption during last year; frequency of condom use in last year (never, sometimes, almost always, and always), recreational drug use during last year, self-perception of HIV knowledge (excellent, good, insufficient, and lack of knowledge), education level; employment status, and previous HIV testing (any in the lifetime). Content of the questionnaire was reviewed by an HIV expert research committee and adjusted according to previous findings from an unpublished pilot study. Then, an in-person counselling session was conducted with each participant.

The pretest counselling (including promotion of condom use), HIV testing, and post-test counselling were conducted using Colombian national guidelines [3]. CLS personnel have been trained in HIV counselling and testing by the *Integrated Program on Health Promotion and Prevention, Diagnosis, and Control of HIV/AIDS*, started in 1988 by the Department of Health Cali, Valle del Cauca, University of Valle, which follows recommendations from the Pan American Health Organization and the World Health Organization [7]. The HIV pretest counselling and the SQ were conducted by professional nurses, social workers, certified laboratory technicians, physicians, and psychologists, for the BAFI-1 strategy and by medicine and nursing students under the supervision of CLS personnel for the BAFI-2 strategy.

The qualitative immunoassay Determine TM HIV-1/2 (Abbot, Abbot Park, IL, USA) was used as screening test for the detection of antibodies to HIV. The performance of this test has been validated in resource-limited settings, where it has shown sensitivity and specificity of 99.8% and 99.4%, respectively [10]. Blood samples were drawn by either a nurse or a certified clinical laboratory technician at the Cali sites and by a clinical microbiologist at the Popayan sites. All personnel were trained directly by Abbot's official laboratory technicians. Collected samples were processed in certified clinical laboratories in Cali and in Popayan.

Test results were available in an average of 5 days. Participants with a positive screening test were contacted, given post-test counselling by CLS personnel, and a second test was performed (a fourth-generation ELISA). If the ELISA was positive, a western blot assay was then performed for confirmation at a national reference laboratory. If the ELISA was negative, the screening test was considered to be false positive. HIV-positive participants were contacted by their health care providers and given post-test counselling.

Follow-up of confirmed cases and access to comprehensive HIV care and antiretroviral treatment were ensured by either linking patients to care in CLS or referring cases to other HIV care programmes according to health insurance affiliation. In-person follow-up was provided to participants seen at CLS and telephone follow-up was provided to participants seen at other clinics.

### 2.6. Performance Indicators

Following the World Health Organization's Guide for monitoring and evaluating national HIV testing and counselling programmes [9] we used the following indicators of process and delivery: (1) rate of positive screening tests, number of positive screening tests/total of participants, (2) rate of return to take confirmatory test, number of participants with positive screening test who returned to take confirmatory test/number of positive screening tests, (3) rate of positive confirmatory tests, number of positive tests by WB/total of participants with WB, (4) HIV prevalence, number of confirmed HIV cases by WB/total population screened, (5) rate of return for medical assessment, number of participants with positive confirmatory test who returned for medical visit/number of positive confirmatory tests, (6) participants who required and initiated ART, and number of participants who required and initiated ART/number of participants with positive confirmatory test who returned for medical consultation.

### 2.7. Ethical Aspects

Participation in the study was completely voluntary, and written informed consent was obtained from each participant, for both HIV testing and the administration of the SQ and counselling. This study was reviewed and approved by the Institutional Review Board of the CLS.

### 2.8. Statistical Analysis

A database using Epi Info Version 3.41 was created. All analyses were conducted using STATA/IC 12.1 (StataCorp LP, Texas, USA). A comparative data analysis between characteristics of the enrolled population and performance indicators of the two BAFI strategies was run using the chi-square and Student's *t*-test.

## 3. Results

The mean age of the participants was 37.2 ± SD 13.5 years in BAFI-1 strategy (ranging from 18 to 89 years old) and 33.5 ± SD 10.2 years in BAFI-2 strategy (ranging from 18 to 64 years old). The main differences between the sociodemographic backgrounds of participants are shown on Table 1. Of note, BAFI-2 participants were more often found to be in stable relationships than BAFI-1 participants, while BAFI-1 participants had higher levels of education and were more consistently health insured than BAFI-2 participants.

Table 2 shows the differences found between both approaches regarding likely risk factors for HIV infection. HIV knowledge and previous HIV testing in both populations were low overall, although participants of BAFI-1 reported a higher self-perception of knowledge (44% versus 28%) and were more likely to have been previously tested for HIV (36% versus 22%). History of previous STIs was high, although participants in BAFI-1 reported fewer infections than those of BAFI-2 (16% versus 21%). Participants in both strategies showed an extremely low proportion of consistent condom use; only 6% and 8% were reported always using condoms in the last year. Self-report of other risk behaviours such as drug use and abuse of alcohol, however, was relatively low (less than 15%) for both populations, and no significant differences were found between them.

Table 3 presents a comparison of the performance between both strategies according to WHO indicators for evaluating HIV testing and counselling campaigns [9]. Several important differences were found between the outcomes of BAFI-1 and BAFI-2. First, BAFI-2 reached a population with higher risk of HIV than BAFI-1, as demonstrated by an HIV prevalence rate 13 times higher than the prevalence rate in BAFI-1 participants (3.86% versus 0.29%). The prevalence in men and women in BAFI-1 was 1.06% and 0%, respectively, and in BAFI-2 was 7.38% and 2.07%, respectively. Second, BAFI-2 achieved 30 percentage points higher in the rate of return for confirmatory test. Interestingly, only 1 in 5 positive BAFI-1 participants required ART initiation, while this was true for 10 out of every 13 BAFI-2 participants, meaning that most BAFI-2 participants were diagnosed late in their infection.

## 4. Conclusions

In both BAFI-1 and BAFI-2, counselling and testing were performed in virtually the same manner and targeted mainly individuals of low-socioeconomic strata. BAFI has demonstrated the ability to offer HIV testing to highly vulnerable populations and proved effective at linking HIV-positive individuals to HIV care and treatment. Although both strategies seem to be effective in reaching people with high-risk factors for HIV, such as those with infrequent condom use and poor HIV knowledge and who have a history of STIs, the collaboration with community leaders to recruit participants in BAFI-2 allowed for the successful engagement of individuals who are the most at risk, people with the lowest education, higher HIV+ confirmed prevalence, and a lower affiliation with health insurance. Interestingly, BAFI-2 engaged a high proportion of women, who have been identified by the United Nations Fund for Population Activities (UNFPA) as increasingly being affected by the epidemic in Colombia [1]. By engaging populations with higher risk of HIV in BAFI-2 we are able to diagnose, engage in care, and treat a significant proportion of individuals who may otherwise have been unaware of their status. In this way we are able to support those living with HIV and have a greater potential to reduce forward transmission [11].

These results are in agreement with previous studies that have demonstrated that offering HIV testing through community leaders or community organizations increases access to HIV testing and counselling for vulnerable populations [12, 13]. Community leaders play an important role in Colombia, as they are knowledgeable of, trusted by, and advocates for their communities. Community leaders in Colombia have traditionally provided basic health education in Tuberculosis and Malaria; yet to date they have not been trained to promote HIV education and testing. Our study suggests that community leaders could effectively promote HIV testing and counselling for men and women at high risk of HIV infection.

One prominent observation was that 61.1% of participants enrolled in BAFI were started on antiretroviral therapy (ART). This means that half of individuals diagnosed with HIV presented late in the course of their infection. At the time of the BAFI implementation the Colombian HIV guidelines recommended treatment only for those with CD4 cell count less than 350 cells/mL. This finding is consistent with previous reports of late diagnosis of HIV in Colombia as well as low access to HIV testing [4].

We found a high proportion of false positives in BAFI-1. We think this was due to the lack of implementing external quality assurance (EQA), which is essential to ensure the accuracy of diagnostic testing [14]. Given that BAFI-1 involved a larger number of participants and health centers in the testing procedure, there was a need for standardized protocols of training and proficiency testing that unfortunately were not considered at the time this study was performed but that are now known to be essential. It is of utmost priority that future studies implement EQA methods such as the “Dried Tube Specimens” method, which has proven to be a simple and effective way to monitor and improve the quality and accuracy of testing in resource-limited settings [14].

The HIV epidemic in Colombia is considered to be concentrated in risk groups such as MSM, transgender populations, and sexual workers [1]. It is important to underline that we are unsure whether the BAFI strategy is suited for studying these high-risk populations, who are consistently reachable with sampling strategies such as respondent-driven sampling [15–17]. Furthermore, we did not collect complete information on gender, sexual orientation, and sexual behaviors to be able to tease out the proportion of MSM and Trans populations in our interventions.

In addition, our study did not measure participation rates as our central interest was on testing our ability to reach certain populations rather than measuring how effective the strategy could be in attracting potential participants. Notably, the starting point in the Colombian context was nil prior to BAFI. BAFI-1 resulted in a substantial number of people not returning for confirmatory tests (37.5%), which is one drawback of this strategy. This was likely due to the fact that results were not available on site and to difficulties in contacting all those who resulted positive in the screening tests.

Despite these limitations, BAFI-1 is an effort to strengthen the capacity of local clinics to lead HIV testing and link positive individuals with HIV care and treatment. Point-of-care testing is not yet available in many private and public clinics in the region for many reasons, including health system-related factors such as insufficient knowledge of HIV counselling strategies, lack of experience with HIV on-site testing and the need to use specialized laboratories, lack of adequate knowledge of HIV care and few health care professionals with expertise in HIV, inconsistent access to antiretroviral medications, high cost of both HIV screening and confirmatory tests, and social barriers such as high levels of stigma against high-risk groups and HIV-positive individuals. Some of these factors were effectively addressed by BAFI as it provided education to health care providers, linked newly diagnosed patients to HIV care programs, guaranteed treatment of positive participants, and provided the test free of cost.

A community-based approach is an optimal choice for future implementations of BAFI in many high-risk groups, such as men who have sex with men, who suffer harsh stigmatization in Colombia. MSM community leaders could be trained to provide HIV counselling and/or could introduce BAFI into venues where MSM gather. Future BAFI campaigns may require training of noncertified laboratory technicians and/or nonprofessional nurses (known in Colombia as *auxiliares de laboratorio* and* auxiliaries de enfermeria, *resp.) to adequately perform point-of-care (POC) HIV testing and counselling (defined as the “provision of a test when the result will be used to make a decision and to take appropriate action, which will lead to an improved health outcome” [18]). Previously, legal requirements have limited HIV testing to be conducted by only a few selected health-care professionals (physicians, professional nurses, and certified clinical laboratory technicians); however, new regulations now permit a wider range of non-professional health care providers to perform HIV testing and counselling provided they receive proper training. This might help to increase access to POC in poor communities, lower the costs associated with POC, and support the sustainability of POC in at-risk communities. It will remain imperative that POC testing carefully adheres to quality control and quality assurance protocols to prevent inaccurate results.

CLS learned key lessons during the implementation of both strategies that will likely help to improve future POC testing. First, rapid screening tests that provide results on site are preferred so results and post-counselling can be given to the tested individuals in one single visit. This will likely further increase the rate of return for confirmatory tests and facilitate personalized assessment of risk factors and tailored postcounselling. Second, blood samples for confirmatory results should be obtained in the first visit along with the screening test sample so that confirmation of positive or negative tests could be performed without the need for a second visit. Third, rapid tests for STIs such as syphilis could be performed in addition to HIV testing, since both our experience and published reports have shown frequent coexistence of syphilis and HIV infection [5, 6].

Over thirty years have passed since beginning of the HIV epidemic; yet the rate of HIV testing in Colombia is just reaching 20% of the at-risk population. This is unacceptably low. Clearly, the expansion of HIV testing is urgently needed. In this regard, the BAFI strategies offer options that promote HIV testing, encourage participation in HIV education, provide free HIV testing and counselling in community settings, and link HIV-positive individuals to health care. This is a unique experience in the Colombian context.

## Figures and Tables

**Table 1:** Demographic characteristics of the participants in BAFI-1 and BAFI-2.

Variables	BAFI-1		BAFI-2		*P* value
	*n* = 2085		*n* = 363	
	*n*	%	*n*	%
Gender					
Male	568	27%	122	34%	0.014
Female	1517	73%	241	66%	
Marital status					
Single/widower/divorced	1098	54%	143	40%	<0.001
Married/cohabitating	953	46%	215	60%	
Educational attainment					
None/primary	780	37%	175	47%	<0.001
Secondary/tertiary	1291	63%	187	53%	
Occupation					
Home	716	35%	118	34%	0.217
Studying	193	9%	35	10%	
Employed	933	46%	174	49%	
Unemployed	211	10%	25	7%	
Affiliated to health insurance	1671	80%	173	48%	<0.001

**Table 2:** Comparison of the risk factors for HIV infection between participants in BAFI-1 and BAFI-2.

Variables	BAFI-1 strategy		BAFI-2 strategy		*P* value
	*n* = 2085		*n* = 363	
	*n*	%	*n*	%
Self-perception of HIV knowledge (self-reported as good or excellent)					
	921	44%	101	28%	<0.001
Previous HIV test	749	36%	81	22%	<0.001
Condom use (always)	176	8%	22	6%	0.144
History of STDs	339	16%	74	21%	0.053
Alcohol abuse (binge drinking, more than 5 drinks in two hours straight)	215	10%	46	13%	0.179
Substance use (recreational drugs)	284	14%	28	8%	0.002
Sexual partners last year (mean ± SD)	1.5 ± 2.4		1.8 ± 5.6		0.3157
Age of first sexual intercourse (mean ± SD)	16.9 ± 3.5		16.1 ± 3.6		0.0001

**Table 3:** Performance indicators for BAFI-1 and BAFI-2 strategies.

	BAFI-1 strategy	BAFI-2 strategy	*P* value
Total of participants	2085	363	
Rate of positive screening tests	1.2% (24/2085)	4.4% (16/363)	<0.001
Rate of return to take confirmatory test	62.5% (15/24)	93.7% (15/16)	0.032
Rate of positive confirmatory tests	40% (6/15)	93.3% (14/15)	0.005
HIV prevalence	0.29% (6/2085)	3.86% (14/363)	<0.001
Rate of confirmed HIV participants return for medical assessment	83.3% (5/6)	92.8% (13/14)	0.521
Rate of confirmed HIV-positive participants who required to initiate ART	20% (1/5)	76.9% (10/13)	0.047
